# 

**Published:** 1884-05

**Authors:** 


					MAY. 1884.
H
AMERICAN JOURNAL
DENTAL SCIENCE.
EDITED BY
F. J. S. GORGAS, M. D., D. D. S.
UOU 18.
THIRD SERIES.
RO; I.
BALTIMORE:
SNOWDEN & COWMAN.
LONDON:
TRUBNER & CO., 60 PATERNOSTER ROW.
1883.
^PAYABLE IN SDYANCEY
PUBLISHED MONTHLY
$2.50 PER RNNUM

				

